# Defining diagnostic thresholds for dissociation between caloric test and vHIT in Ménière’s disease

**DOI:** 10.3389/fneur.2025.1651714

**Published:** 2025-08-19

**Authors:** Tae Uk Cheon, Ju Ha Park, Ji Seop Lee, Seong Hoon Bae

**Affiliations:** Department of Otorhinolaryngology, Gangnam Severance Hospital, Yonsei University College of Medicine, Seoul, Republic of Korea

**Keywords:** Ménière’s disease, vestibular function, caloric test, video head impulse test, diagnostic accuracy

## Abstract

**Objective:**

Ménière’s disease (MD) presents with episodic vertigo, hearing loss, and tinnitus; however, its diagnosis remains challenging owing to symptom overlap with other vestibular disorders. We evaluated the diagnostic value of dissociation between caloric test and video head impulse test (vHIT) results in MD compared to vestibular schwannoma (VS) and benign paroxysmal positional vertigo (BPPV).

**Methods:**

A retrospective analysis included 195 patients with MD (*n* = 51), VS (*n* = 112), or BPPV (*n* = 32). Vestibular function was assessed using caloric tests and vHIT. Dissociation was defined as an abnormal caloric response [canal paresis (CP) > 25%] with a normal vHIT gain (>0.80). Diagnostic accuracy was assessed using diagnostic odds ratio (DOR) and receiver operating characteristic curves.

**Results:**

Dissociation was more frequent in MD (56.9%) than in VS (25.0%) or BPPV (9.4%) (*p* < 0.001). It effectively distinguished MD from BPPV (DOR = 12.74) but was less useful for MD vs. VS (DOR = 3.96). CP differentiated MD from BPPV but not VS.

**Conclusion:**

Dissociation between caloric and vHIT results is a specific indicator of MD, aiding differentiation from BPPV. However, its utility for distinguishing MD from VS is limited.

## Introduction

Ménière’s disease (MD) is a complex, multifactorial syndrome characterized by episodic vertigo, aural fullness or tinnitus, and fluctuating hearing levels (1–3). The Bárány Society, in collaboration with other international academic institutions, has established diagnostic criteria for MD based primarily on clinical presentations and audiometric findings (4). Although vestibular function tests can aid in differentiating MD from other vestibular disorders, they are not essential for diagnosis.

The caloric test, introduced by Bárány, and the head impulse test, developed by Halmagyi and Curthoys, are widely used to assess vestibular function (5, 6). MacDougall et al. later refined the head impulse test into the video head impulse test (vHIT), enabling the detection of covert saccades (7, 8). These tests evaluate semicircular canal function across different frequency ranges: the caloric test assesses very low-frequency stimuli, whereas the vHIT evaluates high-frequency stimuli (9–11).

Although the pathophysiology of MD remains incompletely understood, substantial evidence suggests a strong association with endolymphatic hydrops (EH), a fluid imbalance in the inner ear (12, 13). Clinical symptoms, including vertigo and hearing loss, often manifest after EH reach a considerable degree, supporting the hypothesis that hydrops progression plays a critical role in symptom onset (14, 15).

Recent studies have reported dissociation between caloric and vHIT results in MD, specifically abnormal canal paresis (CP) in caloric testing with normal vHIT gain (10, 16–20). Although this dissociation is frequently observed, a reliable cut-off value for distinguishing MD based on these test results has yet to be established. Additionally, limited studies have assessed the diagnostic effectiveness of dissociation in differentiating MD from other vestibular disorders. Therefore, this study aimed to investigate the dissociation between caloric and vHIT responses in MD compared to horizontal canal benign paroxysmal positional vertigo (HC-BPPV) and vestibular schwannoma (VS). The study further evaluated whether this dissociation is specific to MD and assessed its diagnostic utility.

## Materials and methods

### Participants

Patients diagnosed with definite MD (meeting the 2015 Bárány Society diagnostic criteria and criteria for low-frequency sensorineural hearing loss), VS (confirmed using imaging studies), or HC-BPPV (diagnosed according to the 2015 Bárány Society criteria) who visited Gangnam Severance Hospital were retrospectively included in the study (4, 21). The inclusion criteria were:

1. Patients aged 18 years or older.
2. Patients who underwent both the caloric test and vHIT at their first diagnostic visit between May 1, 2017, and April 30, 2024.

The exclusion criteria were:

1. Bilateral disease (bilateral MD or bilateral VS).
2. Bilateral vestibulopathy, defined as a horizontal canal vHIT gain < 0.6 in both ears or a sum of caloric slow-phase peak velocity < 6°/s in each ear.

Finally, 195 patients were included in the study. The study protocol was approved by the Institutional Review Board of Gangnam Severance Hospital (IRB No. 3–2024-0411), and the requirement for informed consent was waived owing to the retrospective nature of the study.

### Vestibular and hearing function tests

Vestibular function was assessed using the bithermal caloric test and vHIT, with test results from the initial diagnosis used for analysis. For the caloric test, each ear was stimulated with air at 24 and 50°C for 30 s, and eye movements were recorded using an infrared video-based system. The maximum slow-phase velocity of nystagmus was measured after each stimulation, and CP was calculated using the Jongkees formula (22). To accurately localize the lesion, CP values were recorded as negative when the abnormal caloric response was observed in the non-lesioned (contralateral) ear. This adjustment ensured a precise assessment of CP specific to the affected side. Before testing, an ENT specialist examined the eardrum, and testing was not conducted if abnormalities were detected.

For the vHIT, a high-frame-rate video-oculography device (ICS Impulse, Otometrics, Denmark) was used. Patients were instructed to fixate on a target 90 cm away while approximately 20 horizontal head impulses were applied manually with unpredictable timing and direction. The head velocity was maintained between 150 and 200°/s, and the mean vestibulo-ocular reflex gain was automatically calculated as the primary parameter.

Audiological examinations were performed for all MD and VS participants. Pure tone audiometry (PTA) thresholds were measured across frequencies from 125 to 8,000 Hz in a sound-treated booth, and the average pure-tone threshold was defined as the mean of 500, 1,000, and 2,000 Hz thresholds. All vestibular and audiological tests were conducted by experienced audiologists.

### Statistical analysis

A one-way ANOVA with Tukey’s multiple comparisons test was used to analyze age differences among the three groups. The diagnostic effectiveness of dissociation was assessed using the Diagnostic Odds Ratio (DOR), calculated as: (True positives × True negatives)/(False positives × False negatives) = (Sensitivity × Specificity)/(1 − Sensitivity) × (1 − Specificity). A DOR > 8 was considered indicative of good diagnostic performance based on its relationship with the area under the curve (AUC) (23, 24). The Receiver Operating Characteristic (ROC) curve was generated using PRISM 8.0 software, and AUC was classified as follows:

- <0.6 = fail
- 0.6–0.7 = poor
- 0.7–0.8 = fair
- 0.8–0.9 = good
- 0.9–1.0 = excellent

A *p*-value < 0.05 was considered statistically significant. All statistical analyses were conducted using IBM SPSS for Windows, version 20.0 (IBM Corp., Armonk, NY, United States). Data are presented as mean ± standard deviation.

## Results

### Patient characteristics

A total of 195 patients met the inclusion and exclusion criteria and were included in the study (Table 1). Of these, 51 had definite MD, 112 had VS, and 32 had HC-BPPV. There were no significant differences among the groups in terms of mean age, sex ratio, or the proportion of the affected side. However, the average PTA was significantly worse in patients with MD than in patients with VS (*p* = 0.001).

**Table 1:** Demographic data of patients by diagnosis group.

	MD (*n* = 51)	VS (*n* = 113)	BPPV (*n* = 32)
Sex (%)			
Male	20 (39.2)	56 (50.0)	6 (18.8)
Female	31 (60.8)	56 (50.0)	26 (81.2)
*p*-value vs. MD	–	*0.201*	*0.050*
Age, years (SD)	59.9 (14.6)	57.0 (13.9)	57.4 (14.8)
*p*-value vs. MD	–	*0.217*	*0.452*
Affected side PTA, dB (SD)	57.1 (16.7)	43.8 (26.2)	–
*p*-value vs. MD	–	*0.001*	–
Disease sides (%)			
Right	21 (41.1)	54 (47.8)	11 (34.4)
Left	30 (58.8)	58 (52.2)	21 (65.6)
*p*-value vs. MD	–	*0.403*	*0.535*
Mean caloric CP, % (SD)	37.8 (35.9)	38.5 (34.5)	**3.8 (19.1)**
*p*-value vs. MD	–	*0.896*	*< 0.001*
Mean vHIT gain of LSCC (SD)	0.96 (0.12)	**0.84 (0.25)**	0.96 (0.11)
*p*-value vs. MD	–	*< 0.001*	*0.910*

Italicized numbers indicate *p*-values and bold numbers denote significant values. MD, Ménière’s disease; VS, vestibular schwannoma; BPPV, benign paroxysmal positional vertigo; SD, standard deviation; CP, canal paresis; PTA, pure tone audiometry; vHIT, video head impulse test; LSCC, lateral semicircular canal.

### Vestibular function across disease groups

**Figure 1:**
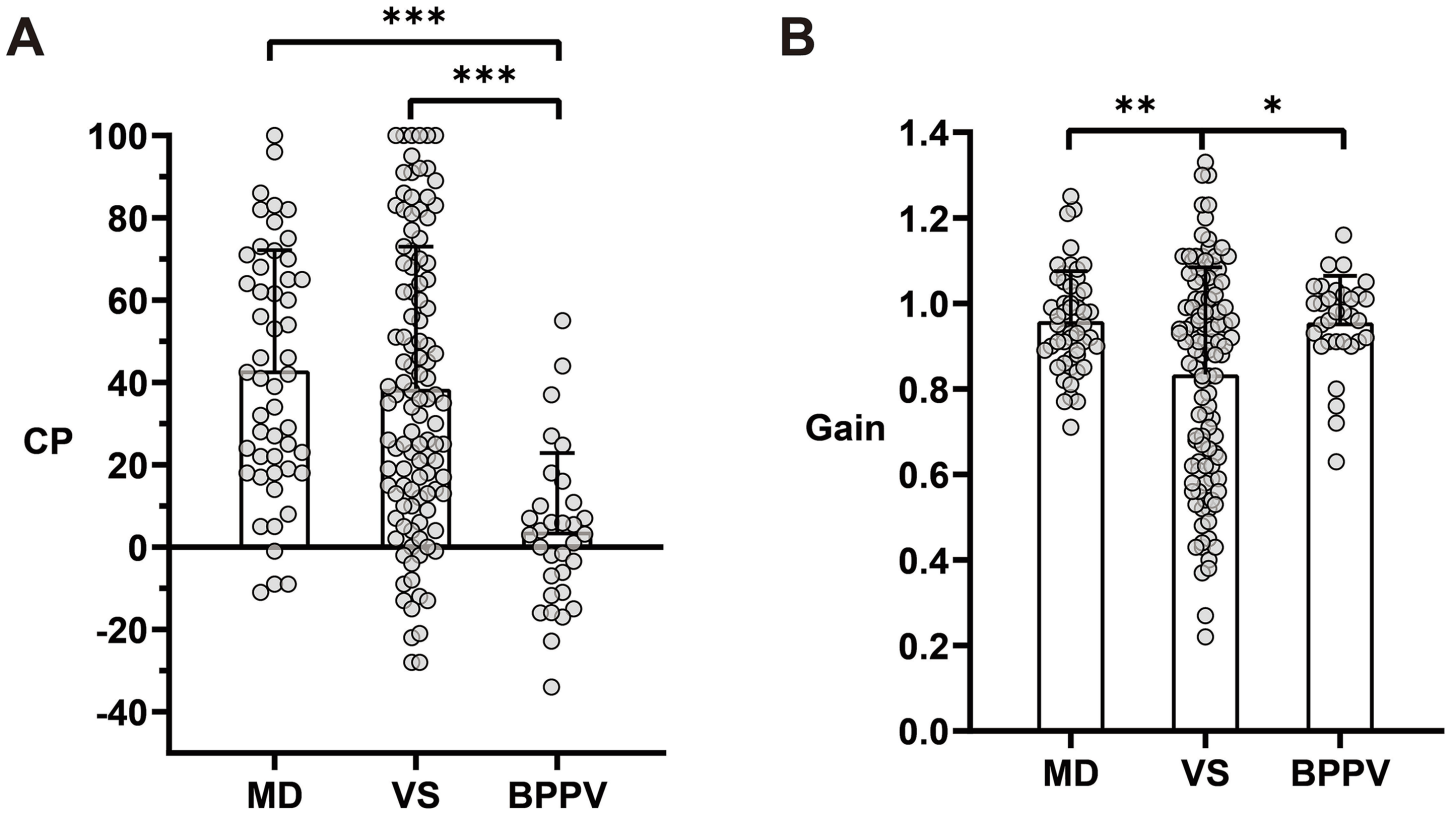
Comparison of caloric CP and vHIT gain among MD, VS, and BPPV groups. **(A)** Significant differences were observed between the MD and VS groups (*p* < 0.001) and between the MD and BPPV groups (*p* < 0.001). **(B)** Significant differences were observed between the MD and VS groups (*p* < 0.01) and between the VS and BPPV groups (*p* < 0.05). Individual data points are represented as dots, with error bars indicating standard deviations. Asterisks denote significance levels: *p < 0.05, **p < 0.01, ***p < 0.001. CP, canal paresis; vHIT, video head impulse test; MD, Ménière’s disease; VS, vestibular schwannoma; BPPV, benign paroxysmal positional vertigo.

The mean CP percentage in caloric tests and the mean vHIT gain of the horizontal semicircular canals in affected ears differed significantly among the three groups (Table 1; Figure 1). Vestibular function was mostly normal in the HC-BPPV group compared to the MD and VS groups. CP was significantly lower in the HC-BPPV group than in the MD (3.8% vs. 37.8%, *p* < 0.001) and VS (3.8% vs. 38.5%, *p* < 0.001) groups. Meanwhile, vHIT gain was significantly lower in the VS group than in the MD (0.834 vs. 0.958, *p* < 0.001) and HC-BPPV (0.834 vs. 0.955, *p* < 0.001) groups.

**Figure 2:**
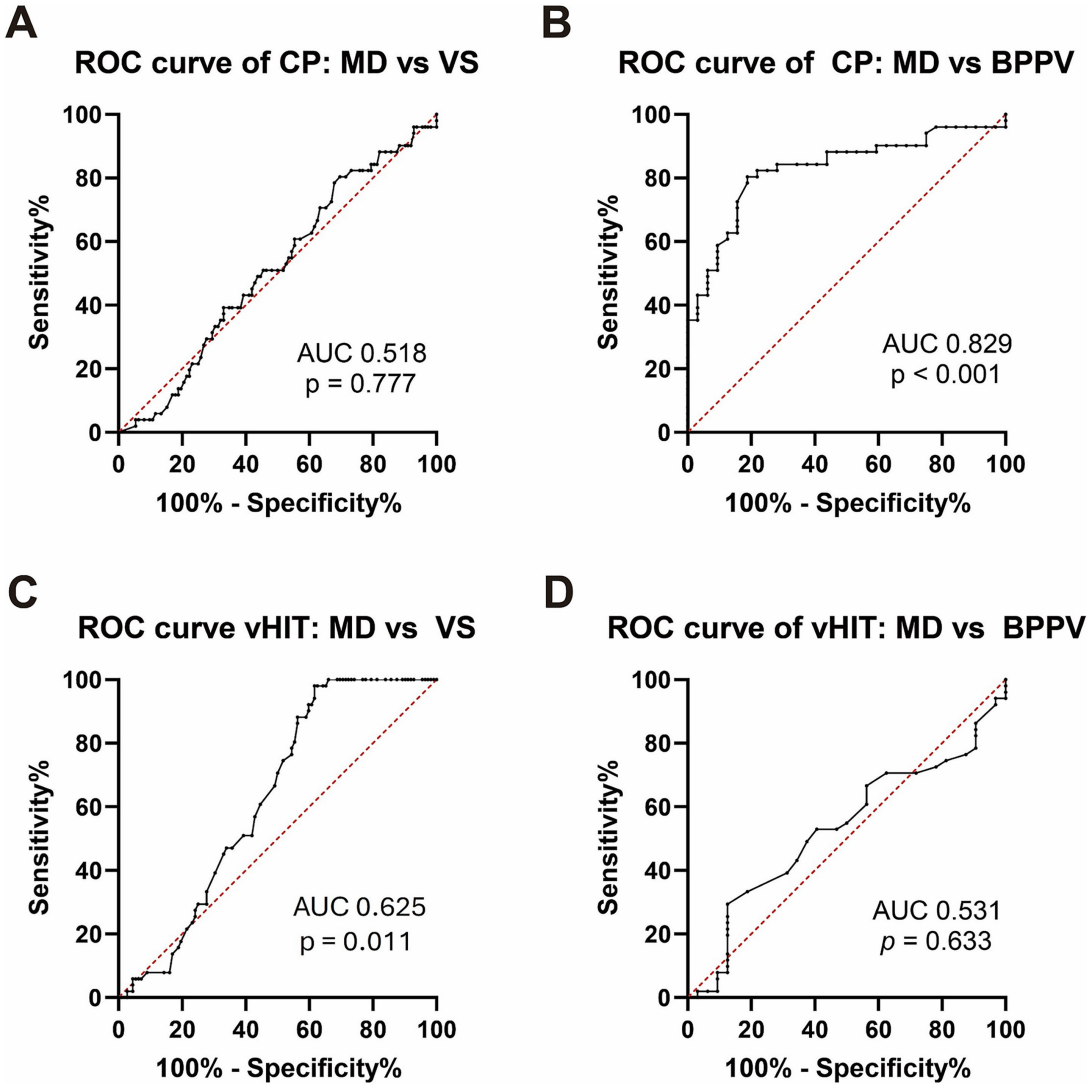
ROC curves for caloric CP and vHIT gain. **(A)** ROC curve for caloric CP distinguishing MD from VS. **(B)** ROC curve for caloric CP distinguishing MD from BPPV. **(C)** ROC curve for vHIT gain distinguishing MD from VS. **(D)** ROC curve for vHIT gain distinguishing MD from BPPV. AUC: area under the curve; ROC, receiver operating characteristics; CP, canal paresis; vHIT, video head impulse test; MD, Ménière’s disease; VS, vestibular schwannoma; BPPV, benign paroxysmal positional vertigo.

ROC curve analysis (Figure 2) showed that CP was a good parameter for distinguishing MD from BPPV (AUC = 0.850, *p* < 0.001); however, it was ineffective for differentiating MD from VS (AUC = 0.539, *p* = 0.423). Conversely, vHIT demonstrated poor accuracy in distinguishing MD from VS (AUC = 0.625, *p* = 0.011) and failed to differentiate MD from BPPV (AUC = 0.531, *p* = 0.633).

### Diagnostic effectiveness of dissociation

Dissociation was defined as an abnormal caloric response (CP > 25%, based on laboratory data) with a normal vHIT gain of the lateral semicircular canal (gain > 0.80) (17, 25–28). In the MD group, 60.8% of patients had abnormal caloric responses; in contrast, 92.2% had normal vHIT results, leading to a dissociation rate of 56.9% (Table 2). The dissociation rate was significantly higher in patients with MD than in those with VS (25.0%, *p* < 0.001) or those with BPPV (9.4%, *p* < 0.001).

**Table 2:** Diagnostic effectiveness of dissociation between caloric test and vHIT.

	MD (*n* = 51)	VS (*n* = 112)	BPPV (*n* = 32)
Caloric test > 25 (%)	31 (60.8)	64 (57.1)	**4 (12.5)**
*p*-value vs. MD	–	*0.662*	*< 0.001*
vHIT > 0.80 (%)	47 (92.2)	**67 (59.8)**	28 (87.5)
*p*-value vs. MD	–	*< 0.001*	*0.705*
Dissociation^a^ (%)	29 (56.9)	**28 (25.0)**	**3 (9.4)**
*p*-value vs. MD	–	*< 0.001*	*< 0.001*
DOR^b^ of dissociation for MD	–	3.96	12.74
Sensitivity for MD	–	56.9%	56.9%
Specificity for MD	–	75.0%	90.6%

Italicized numbers indicate *p*-values and bold numbers denote significant values. ^a^CP > 25 and vHIT gain > 0.80. ^b^The diagnostic odds ratio (DOR) was calculated using the equation: (True positives × True negatives)/(False positives × False negatives) and was considered significant if DOR > 8. MD, Ménière’s disease; VS, vestibular schwannoma; BPPV, benign paroxysmal positional vertigo; CP, canal paresis; vHIT, video head impulse test.

The diagnostic effectiveness of dissociation was assessed using DOR. Dissociation had a DOR of 3.96 for distinguishing MD from VS and 12.74 for distinguishing MD from BPPV. Sensitivity and specificity were 56.9 and 75.0% for MD vs. VS and 56.9 and 90.6% for MD vs. BPPV.

## Discussion

This study demonstrated that dissociation—defined as CP > 25% with a vHIT gain > 0.80—was significantly more frequent in patients with MD (56.9%) compared to those with VS (25.0%) and BPPV (9.4%). The diagnostic effectiveness of dissociation for distinguishing MD from BPPV was good, with a sensitivity of 56.9%, a specificity of 90.6%, and a DOR of 12.7. However, its effectiveness for differentiating MD from VS was limited, with a DOR of 3.96, a sensitivity of 56.9%, and a specificity of 75.0%.

Dissociation between caloric and vHIT responses has been increasingly recognized as a clinically relevant phenomenon in MD. Several key studies have established this dissociation as not only common but also potentially specific to MD (17, 19, 25). Multiple comparative studies have confirmed that dissociation is significantly more frequent in MD than in other vestibular disorders such as vestibular migraine, BPPV, or vestibular neuritis. This dissociation pattern—abnormal caloric responses with preserved vHIT—has been repeatedly reported in MD cohorts but is rare in other peripheral or central disorders (29–32).

Histopathological studies of human temporal bones have established a strong correlation between MD and EH (12, 13). The caloric test, a key vestibular assessment tool, operates differently in patients with EH compared to those without. It functions by applying a thermal stimulus to the external auditory canal, which is transmitted to the lateral semicircular canal—the closest semicircular canal to the tympanic membrane. This process creates a temperature gradient between the two arms of the canal, leading to a density difference in the endolymph. The resulting hydrostatic pressure deflects the cupula, stimulating the hair cells and generating an eye velocity response (5, 33, 34). However, in EH, the membranous duct of the lateral semicircular canal expands, increasing its cross-sectional area. This expansion facilitates localized endolymphatic flow during caloric stimulation, dissipating the hydrostatic pressure typically induced by the thermal gradient. Consequently, cupular stimulation is reduced, leading to a diminished caloric response compared to a normal labyrinth (19).

In contrast, vHIT results in patients with EH remain largely unaffected (35). Unlike the low-frequency caloric test (0.003 Hz), vHIT assesses vestibular function at a physiological frequency (approximately 2.5 Hz), corresponding to the semicircular canals’ natural response to angular acceleration during typical head movements (9, 36). vHIT does not rely on static conditions; hence, the structural expansion of the membranous duct in EH has minimal impact, allowing for normal vHIT outcomes (37). Notably, the reverse dissociation pattern—normal caloric responses with impaired vHIT—may suggest central vestibular involvement. In fact, approximately half of such cases have been reported to result from central lesions such as cerebellar or brainstem pathology (38).

Some studies suggest that dissociation between caloric test and vHIT results in MD may be attributed to the vulnerability of type II hair cells in MD (27, 39). A related hypothesis proposes that type I hair cells primarily respond to high-frequency stimuli (vHIT); in contrast, type II hair cells respond to low-frequency stimuli (caloric test), potentially explaining the observed dissociation. However, more recent findings indicate that both type I and type II hair cells are equally affected in MD, challenging this frequency-dependent theory (40). Additionally, the complex relationships between hair cells and afferent neurons, along with fluctuating test results in early MD, weaken the explanatory power of this hypothesis (41). Instead, structural changes such as EH provide a more plausible explanation for the dissociation.

The dissociation between caloric and vHIT results has been repeatedly reported in previous studies (16, 17, 19, 20, 25, 40). However, there is a lack of studies that have systematically explored the optimal threshold values to define dissociation between caloric and vHIT results for the differential diagnosis of various vestibular disorders. While the findings are promising, the retrospective cross-sectional design limits the ability to assess longitudinal changes in vestibular function. Additionally, although patients with BPPV were included as a control group, they do not represent truly healthy controls. Future research would benefit from larger cohorts of patients with MD and other dizziness-related disorders, as well as the inclusion of healthy volunteers.

## Conclusion

This study demonstrated that the dissociation between caloric test and vHIT results is a specific finding in patients with MD. The diagnostic effectiveness of dissociation was good for distinguishing MD from BPPV but limited for differentiating MD from VS. Clinically, screening for dissociation between caloric and vHIT results in patients with dizziness may provide a useful clue for suspecting MD.

## Data Availability

The raw data supporting the conclusions of this article will be made available by the authors, without undue reservation.
